# "Glucose and ethanol-dependent transcriptional regulation of the astaxanthin biosynthesis pathway in *Xanthophyllomyces dendrorhous*"

**DOI:** 10.1186/1471-2180-11-190

**Published:** 2011-08-23

**Authors:** Andrés Marcoleta, Mauricio Niklitschek, Aniela Wozniak, Carla Lozano, Jennifer Alcaíno, Marcelo Baeza, Víctor Cifuentes

**Affiliations:** 1 Departamento de Ciencias Ecológicas y Centro de Biotecnología, Facultad de Ciencias, Universidad de Chile, Las Palmeras 3425, Ñuñoa, Santiago, 7800024, Chile

## Abstract

**Background:**

The yeast *Xanthophyllomyces dendrorhous *is one of the most promising and economically attractive natural sources of astaxanthin. The biosynthesis of this valuable carotenoid is a complex process for which the regulatory mechanisms remain mostly unknown. Several studies have shown a strong correlation between the carbon source present in the medium and the amount of pigments synthesized. Carotenoid production is especially low when high glucose concentrations are used in the medium, while a significant increase is observed with non-fermentable carbon sources. However, the molecular basis of this phenomenon has not been established.

**Results:**

In this work, we showed that glucose caused transcriptional repression of the three genes involved in the synthesis of astaxanthin from geranylgeranyl pyrophosphate in *X. dendrorhous*, which correlates with a complete inhibition of pigment synthesis. Strikingly, this regulatory response was completely altered in mutant strains that are incapable of synthesizing astaxanthin. However, we found that addition of ethanol caused the induction of *crtYB *and *crtS *gene expression and promoted *de novo *synthesis of carotenoids. The induction of carotenogenesis was noticeable as early as 24 h after ethanol addition.

**Conclusion:**

For the first time, we demonstrated that carbon source-dependent regulation of astaxanthin biosynthesis in *X. dendrorhous *involves changes at the transcriptional level. Such regulatory mechanism provides an explanation for the strong and early inhibitory effect of glucose on the biosynthesis of this carotenoid.

## Background

Astaxanthin (3,3'-dihydroxy-β,β-carotene-4,4'-dione) is a red-orange carotenoid pigment of high commercial interest, mainly because of its use as a dietary additive in the aquaculture industry [1,2] and its many benefits to human health [3]. As further properties of this carotenoid have been discovered, demand has increased significantly, thus motivating the identification of new sources of the pigment as an alternative to its chemical synthesis. One of the most promising natural sources of astaxanthin is the basidiomycete yeast *Xanthophyllomyces dendrorhous*. This yeast normally produces the pigment in its natural environment, probably to protect itself from other chemical compounds. Carotenoids are potent antioxidants, and the main function of astaxanthin in *X. dendrorhous *has been proposed to be protection against reactive oxygen species and accompanying cellular damage. This hypothesis is supported by the observations that *X. dendrorhous *is particularly susceptible to some activated oxygen species, has low levels of catalase activity and possesses Mn-superoxide dismutase but not other classes of superoxide dismutases that are present in many other yeasts [4].

The biosynthesis of astaxanthin from the isoprenoid precursor geranylgeranyl pyrophosphate (GGPP) in *X. dendrorhous *requires at least four enzymatic activities, which are catalyzed by enzymes encoded by the genes *crtI, crtYB *and *crtS*. During the first phase of biosynthesis, the phytoene synthase activity of the bifunctional enzyme phytoene-β-carotene synthase (PBS, product of *crtYB*) catalyzes the condensation of two GGPP molecules to produce one molecule of phytoene, the first carotenoid of the pathway [5]. After four desaturation reactions catalyzed by the enzyme phytoene desaturase (product of *crtI*), phytoene is transformed into a lycopene [6]. Subsequently, the lycopene is cyclized to form β-carotene via the β-carotene synthase activity of PBS [5]. Finally, the β-carotene is oxidized at both ends in a process that requires cytochrome p450 astaxanthin synthase (product of *crtS*) [7,8]. This reaction requires the accessory activity of a cytochrome p450 reductase enzyme as an electron donor [9].

Although the structural genes needed for the synthesis of astaxanthin in this yeast have been characterized, the regulatory mechanisms that control this process are largely unknown. Importantly, alternative processing of *crtYB *and *crtI *have been reported to occur [10], although the implications of this phenomenon have not been established. In addition, alternative transcripts of both genes have several premature stop codons in all three reading frames, and they likely encode non-functional proteins [10].

*X. dendrorhous *can develop two metabolic modes depending on the type of carbon source present in the medium. Glucose or other fermentable sugars are assimilated through the glycolytic pathway followed by alcoholic fermentation to produce ethanol, even in the presence of oxygen [11]. However, non-fermentable carbon sources, such as ethanol or succinate, are transformed to acetyl-CoA and are processed through the citric acid cycle. Thus, energy is produced mainly through oxidative phosphorylation.

There is a strong correlation between the carbon source used and the level of pigment synthesized; high glucose concentrations as the carbon source yield minimal pigment synthesis [12,13], ethanol as the carbon source yields greater pigment synthesis [14]. In addition, when *X. dendrorhous *is grown on glucose as the only carbon source, the induction of carotenogenesis coincides with sugar depletion and the beginning of ethanol consumption (produced by fermentation of the carbohydrate) [15]. Finally, previous studies have reported the presence of putative MIG1 binding sites in the promoter regions of the *crtYB*, *crtI *and *crtS *genes [7]. MIG1 was originally described in *S. cerevisiae *and is a well-known transcription factor that mediates glucose-driven transcriptional repression processes in various yeasts [16-19].

Taken together, this information suggests that glucose and ethanol may modulate carotenoid biosynthesis by regulating the expression of the carotenogenesis genes. In this study, we characterized the effect of glucose and ethanol on the expression of *crtYB*, *crtI *and *crtS *and on the early stages of carotenoid production.

## Results

### Effect of glucose on the expression of carotenoid biosynthesis genes

Several observations support the hypothesis that glucose has an inhibitory effect on carotenoid production in *X. dendrorhous*. Among other findings, the discovery of potential MIG1-binding sites in the promoter regions of several carotenogenic genes suggests that transcriptional regulation mechanisms may be involved in this inhibition. To determine whether glucose affects the expression of the carotenogenic genes, *X. dendrorhous *cells were grown in YM liquid medium without glucose to prevent the production of ethanol, which can influence the phenomenon under investigation. Once the culture reached stationary phase (optical density between 3.5 and 4), it was divided in two Erlenmeyer flasks, one of which had glucose added to a final concentration of 20 g/l (the concentration normally used in most media), while the other flask was left untreated (control). Both aliquots were incubated at 22°C with constant swirling, and cell samples were taken 0, 2, 4, 6 and 24 h after the addition of glucose. From these samples, total RNA was extracted and the expression of several genes was determined relative to control using quantitative RT-PCR.

To validate our experimental approach, we first measured the effect of glucose on the expression of genes normally regulated by glucose in related yeasts. As a glucose repression control, we used a genomic sequence from *X. dendrorhous *called *glucose repressible gene 2 *(*grg2*) [GenBank: JN043364]. This gene is highly repressed by glucose in *N. crassa *and in many other yeasts [20,21]. As a glucose induction control, we used the pyruvate decarboxylase gene *PDC*, which is induced by glucose in several fungi and yeasts [22-25]. For this experiment, genomic *PDC *and its cDNA were sequenced, its intron-exon structure was determined and its sequence was deposited in the database [GenBank: HQ694557 and HQ694558].

**Figure 1:**
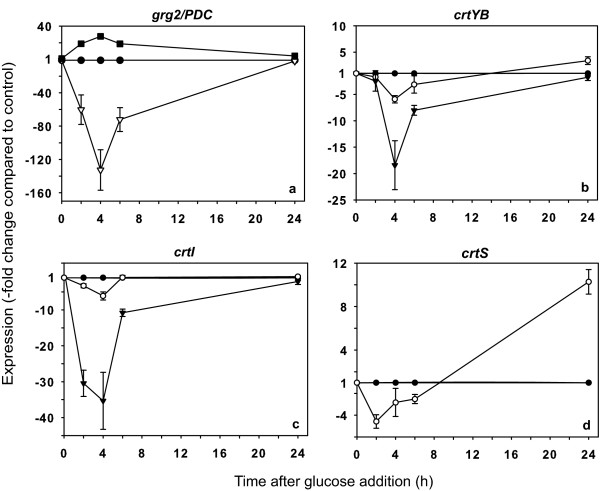
**Effect of glucose on expression of the carotenoid biosynthesis genes in *X. dendrorhous***. The gene expression kinetics in the wild-type strain after adding glucose (20 g/l final concentration) was determined with respect to the control (black circle) for the carotenogenesis genes and for the *grg2 *and *PDC *genes. a: *grg2 *(white inverted triangle) and *PDC *(black square); b: mm*crtYB *(white circle) and am*crtYB *(black inverted triangle); c: mm*crtI *(white circle) and am*crtI *(black inverted triangle); d: *crtS *(white circle). The error bars correspond to the standard deviation (n = 3). The negative values on the y-axis denote decreases relative to the control. mm: mature transcript, am: alternative transcript.

By evaluating the expression of the genes mentioned above, we found that the addition of glucose caused an approximately 130-fold decrease in the mRNA levels of the *grg2 *gene and an approximately 28-fold increase in the mRNA levels of the *PDC *gene (Figure 1a). Both effects reached their maximums 4 h after the addition of the carbohydrate and were not detectable after 24 h.

After validating the experimental approach, we characterized the effect of glucose on the expression of the *crtYB*, *crtI *and *crtS *genes. The mRNA levels of the three carotenogenic genes decreased considerably upon the addition of glucose. In the case of the *crtYB *gene (Figure 1b), both the mature and the alternative transcripts reached minimum levels 4 h after the addition of glucose and returned to basal levels within 24 h. Curiously, the effect of glucose was significantly greater on the alternative messenger (~18-fold decrease) than on the mature messenger (~6-fold decrease). This result is striking, considering that both messengers are transcribed from the same promoter. A similar effect occurred with the *crtI *gene (Figure 1c); glucose decreased the levels of the alternative mRNA (~35-fold decrease) to a greater extent than the mature mRNA (~6-fold decrease). The repression effect disappeared quickly for both transcripts and was not detectable 24 h after the addition of glucose. In the case of the *crtS *gene (Figure 1d), glucose had a smaller effect, with an approximately 5-fold decrease in the mRNA levels at 2 h after treatment. Interestingly, 24 h after adding the sugar, expression of the *crtS *gene was increased 10-fold.

**Figure 2:**
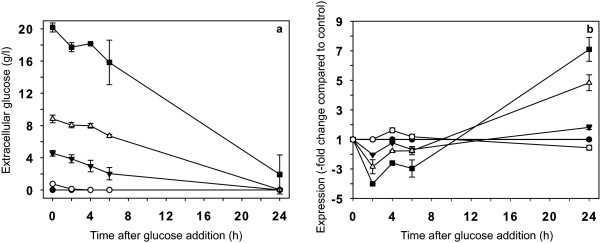
**Dose-response effect of glucose-mediated transcriptional repression of the *crtS *gene**. Cultures of UCD 67-385 were grown until reaching stationary phase and were divided into five aliquots. Glucose was added to each aliquot to a final concentration of 20 (black square), 10 (white triangle), 5 (black inverted triangle) or 1 g/l (white circle); no glucose was added to the control culture (black circle). Subsequently, the amount of glucose remaining in the media was determined (a), along with the relative expression of the *crtS *gene (b) at 2, 4, 6 and 24 h post-treatment. The error bars correspond to standard deviation (n = 3). The negative values on the y-axis denote decreases relative to the control.

Depletion of the glucose added to the medium and the subsequent decrease of the repression effect caused by glucose may be responsible for the quick return of the mRNAs to their basal levels. To evaluate this possibility, we determined the amount of glucose remaining throughout the 24-h-period during which the expression response was observed (Figure 2a). The results indicated that the kinetics of glucose consumption were much slower than the return of the mRNAs to their basal levels. For most of the genes studied, the glucose response (20 g/l final concentration) occurred mainly during the first 6 h after treatment. However, during that time frame, only 20% of the glucose was consumed, with approximately 16 g/l remaining in the medium. Given this observation, we next determined whether lower concentrations of glucose were capable of generating a repression response. We determined the relative expression of the carotenogenesis genes after adding glucose to final concentrations of 10, 5 and 1 g/l. For all of the genes assayed (Figure 2b, only data for *crtS *is shown as an example), we observed that the maximum repression effect increased as the glucose concentration was increased. However, the response kinetics was practically identical for all of the glucose concentrations analyzed. The repressive effects on the carotenogenic genes were not detectable when using a glucose concentration of 1 g/l, suggesting that the response threshold is more than 1 g/l. The return of the carotenogenic gene expression to basal levels appeared to be independent of the amount of glucose remaining in the culture medium, as the kinetics of the transcriptional response did not vary upon changing the initial concentration of glucose added. To further analyze this observation, the concentration of extracellular glucose was determined at different times for all of the initial sugar concentrations studied (Figure 2a). We observed that greater than 50% of the initial glucose remained in all cases 6 h after the addition of glucose. Thus, once the glucose had caused a decrease in the mRNA levels, recovery to the original expression levels was not completely dependent on the amount of glucose remaining in the culture medium.

### Effect of ethanol on the expression of carotenogenesis genes

**Figure 3:**
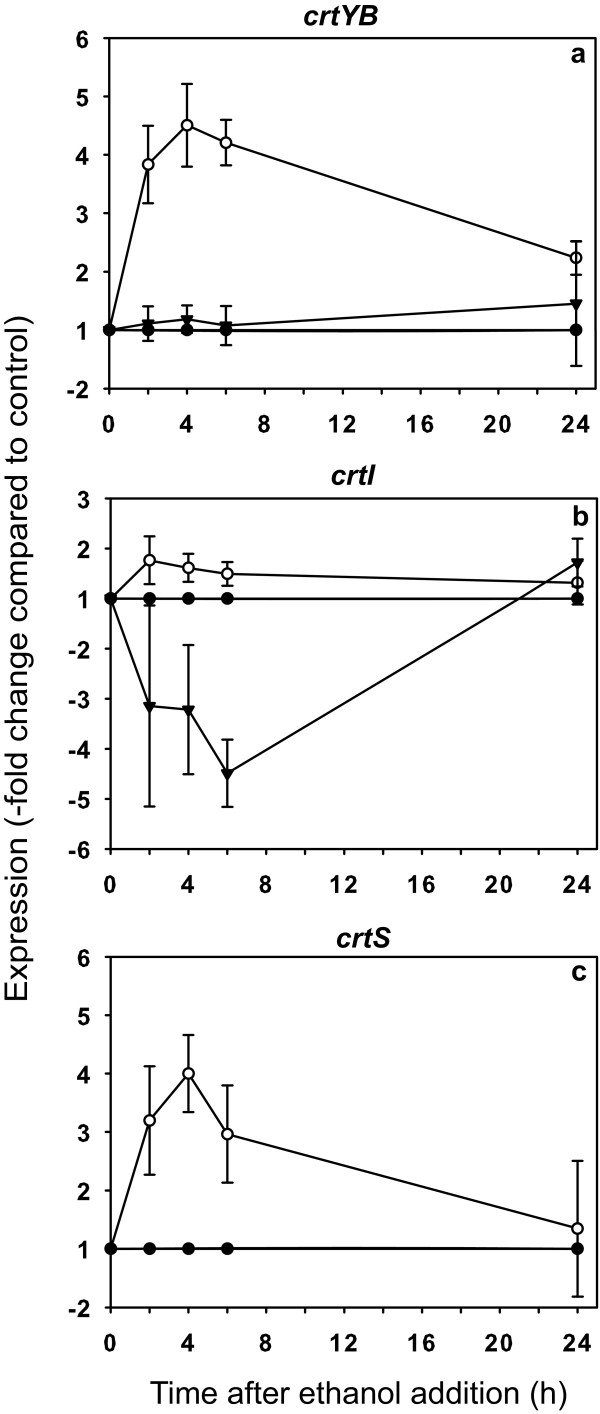
**Effect of ethanol on expression of the carotenogenesis genes**. The expression kinetics after adding ethanol (2 g/l final concentration) was determined relative to control (black circle) for the *crtYB *mature mRNA (*mm*, white circle) and the alternative mRNA (*am*, black inverted triangle) (a); *mmcrtI *and *amcrtI *(b); and *crtS*(c). The error bars correspond to standard deviation (n = 3). The negative values on the y-axis denote decreases relative to control.

Previous reports indicated that adding ethanol to *X. dendrorhous *cultures increased the amount of pigments produced after five days [14,26]. In addition, when the yeast was grown with glucose as the only carbon source, the induction of carotenogenesis coincided temporally with the depletion of the glucose and the maximum concentration of ethanol (~2 g/l) produced by fermentation of the sugar [15]. Ethanol may upregulate the expression of the carotenogenic genes, thus inducing carotenoid production. To test this possibility, we used an experimental design similar to that of the glucose experiments, but we added ethanol instead of glucose to a final concentration of 2 g/l. The results indicated that upon the addition of ethanol, there was an approximately 4.5-fold increase in the levels of the mature messenger of *crtYB*, but there was no significant effect on expression of its alternative version (Figure 3a). Ethanol did not have a significant effect on the expression of the mature messenger of the *crtI *gene, but it caused up to a 4.5-fold decrease in the expression of the alternative transcript, which returned to basal levels after 24 h (Figure 3b). Finally, the addition of ethanol caused up to a 4-fold increase in the mRNA levels of the *crtS *gene, which reached its maximum induction level 4 h after treatment (Figure 3c). These results indicate that the addition of ethanol (2 g/l) caused a significant increase in the expression of two of the three genes required for the synthesis of astaxanthin from GGPP in *X. dendrorhous*. This phenomenon could explain, at least in part, the induction of carotenoid production upon ethanol addition.

### Effect of glucose and ethanol on synthesis of pigments

**Figure 4:**
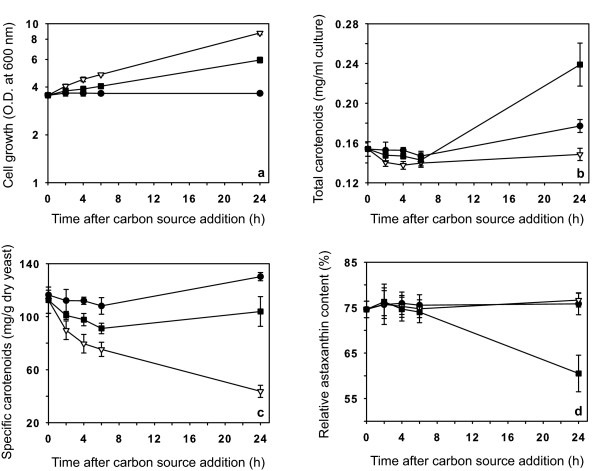
**Effect of glucose and ethanol on cell growth and early production of pigments in *X. dendrorhous***. Cell growth (a), total amount of carotenoids produced by culture volume (b) and carotenoids produced by biomass (c) were determined for the control (untreated, black circle) and cultures treated with glucose (20 g/l final, white inverted triangle) or ethanol (2 g/l final, black square). In addition, the relative content of astaxanthin with respect to the total amount of carotenoids detected in each sample was determined (d). The error bars correspond to standard deviation (n = 3).

To address the biological significance of the changes in the mRNA levels of the carotenogenesis genes upon glucose and ethanol addition, we tested the effect of these compounds on early pigment production. For this experiment, we measured carotenoid production during a short time after the carbon source addition, thus allowing a more direct correlation between both phenomena. For this purpose, *X. dendrorhous *cells were grown in YM medium without glucose for up to 24 h after the stationary phase had been reached, at which point the cultures were divided into three aliquots. Glucose was added to one of the aliquots to a final concentration of 20 g/l. Ethanol was added to another aliquot to a final concentration of 2 g/l and the remaining aliquot was left untreated (control). Subsequently, aliquots from these cultures were collected 2, 4, 6 and 24 h after treatment, and the biomass production as well as the amount and composition of carotenoids present in each sample were determined. We found that the addition of glucose caused an increase in biomass that was notably higher than that observed 24 h after the addition of ethanol (Figure 4a). However, analysis of the total amount of carotenoids per ml of culture (Figure 4b) revealed that no pigments were produced even 24 h after adding the carbon source in the glucose-treated aliquot. By contrast, upon addition of ethanol, there was an almost 1.8-fold increase in the amount of carotenoids present 24 h after treatment as compared with control (Figure 4b). In this case, although there was also an increase in biomass, the increase was coupled with pigment production. By analyzing the specific amount of carotenoids, we found that glucose addition caused a progressive decrease in the amount of pigments produced per dry biomass unit (ppm) (Figure 4c). This decrease became noticeable just 2 h after the addition of the sugar, reaching a level that was three-fold less than in the control after 24 h, and was mainly due to the increase in biomass and lack of pigment synthesis. However, upon the addition of ethanol, the amount of carotenoids per unit of biomass remained relatively constant, reaching a level slightly lower than the control 24 h after the carbon source was added.

Previous studies performed in our laboratory indicated that as *X. dendrorhous *cultures age, the proportion of carotenoid intermediates relative to astaxanthin decreases. This phenomenon is accompanied by an increase in the relative amount of astaxanthin, which was explained by the termination of the *de novo *synthesis of pigments and the conversion of all of the intermediates to the final product of the pathway. Therefore, *de novo *synthesis of pigments can be evaluated by determining the proportion of intermediates relative to the amount of the final product (astaxanthin) over the course of the experiment. Accordingly, an analysis of the composition of the carotenoids present in the previously analyzed samples was conducted using reverse phase liquid chromatography (RP-HPLC). We measured the relative content of astaxanthin with respect to the total amount of pigments detected in each sample (*i.e*., astaxanthin, phoenicoxanthin, canthaxanthin, 3-OH-ketotorulene, echinenone, 3-OH-echinenone, neurosporene and β-carotene) (Figure 4d). In the control condition, the amount of astaxanthin remained constant at approximately 75% over the 24-h period studied, indicating that there were no intermediates generated. A very similar situation was observed when glucose was added; the proportion of astaxanthin remained the same as in the control at each of the times analyzed. A completely different phenomenon was observed when ethanol was added to the medium. In this case, 24 h after the addition of the carbon source, a significant decrease in the relative amount of astaxanthin was observed. This observation can be explained by the generation of carotenoid intermediates as a result of the induction of pigment biosynthesis. These results indicate that the addition of ethanol caused an increase in the amount of total carotenoids by promoting the *de novo *synthesis of pigments. In contrast, when glucose was added to the medium, there was an inhibition of pigment synthesis that was maintained over the entire analyzed time period. Importantly, both effects were detectable as early as 24 h after the addition of the carbon source and the effects correlated temporally with changes in the mRNA levels of the carotenogenesis genes.

### Effect of glucose on the expression of carotenogenesis genes in mutant strains incapable of synthesizing astaxanthin

**Figure 5:**
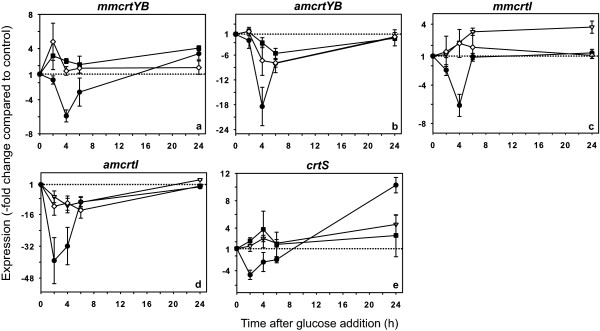
**Effect of glucose on the expression of carotenogenesis genes in mutant strains incapable of synthesizing astaxanthin**. Strains: T-YBHH2 (*crtYB*-/-, white inverted triangle), T-I21H1H (*crtI*-/-, black square) and T-SHH2 (*crtS*-/-, white diamond). Levels of mRNA for mature *crtYB *(a), alternative *crtYB *(b), mature *crtI *(c), alternative *crtI *(d) and *crtS *(e) in glucose-treated cultures (20 g/l) were determined for each strain relative to the control. The values from wild-type strain UCD-67 385 (*wt*, black circle) were taken from Figure 1 and are included here as a reference. The dotted line corresponds to the expression value in the control condition. The error bars correspond to standard deviation (n = 3). The negative values on the y-axis denote decreases relative to the control.

The results described above demonstrate that the inhibition of *de novo *synthesis of pigments caused by high glucose concentrations parallels the decrease in the expression levels of all of the genes involved in carotenogenesis. However, previous reports have indicated that carotenogenesis may be regulated by some type of feedback mechanism, by which the relative proportion of astaxanthin regulates the total amount of carotenoids synthesized [27]. Given our observations, the feedback mechanism mediated by astaxanthin in the carotenoid biosynthesis pathway may involve regulatory mechanisms at the transcriptional level, and the presence and composition of pigments may affect the transcriptional response triggered by the addition of glucose to the medium. To test this hypothesis, we performed glucose addition experiments using homozygous mutant strains that are incapable of synthesizing astaxanthin. The strains used were T-YBHH2 (*crtYB^-^*), T-I21H1H (*crtI*^-^) and T-SHH2 (*crtS^-^*), as described in a previous work [28]. First, we determined that the response of *grg2 *and *PDC *expression to glucose was similar in all of the strains studied and did not depend on the synthesis of pigments (data not shown). In contrast, different results were observed for the mutants of the carotenogenesis pathway genes. For the *crtYB *gene, the 6-fold repression of mature messenger observed in the wild-type strain in response to glucose completely disappeared and was replaced by a slight induction in both, the mutant that accumulates phytoene (*crtI*^-^) and the mutant that accumulates β-carotene (*crtS^-^*) (Figure 5a). However, the levels of alternative transcripts in both mutant strains exhibited a glucose-mediated decrease that was less than the one observed in the wild-type strain (Figure 5b). A similar phenomenon was observed for the *crtI *gene; both, the mutant incapable of synthesizing carotenoids (*crtYB^-^*) and the mutant that accumulates β-carotene (*crtS^-^*) showed a complete lack of glucose repression of the mature transcript (Figure 5c) and a very diminished response of the alternative transcript levels (Figure 5d). Finally, in the case of the *crtS *gene, the slight repression by glucose observed in the wild-type strain was replaced by a slight induction in the *crtYB^- ^*and *crtI^-^*mutants (Figure 5e). These results indicate that the expression of the *crtYB*, *crtI *and *crtS *genes in response to the addition of glucose depends, at least in part, on the normal synthesis of astaxanthin or on the presence of this compound in the cell.

## Discussion

Carotenogenesis in *X. dendrorhous *is a complex process with regulatory mechanisms that have not been fully elucidated. Several studies have reported that the amount and composition of carotenoids may be greatly modified depending on the carbon source used [12-14,29,30]. A common observation is that the synthesis of pigments is particularly low at glucose concentrations greater than 15 g/l [12,13,31]. However, until this study, there was no available data on how glucose exerts its repressive effect on carotenogenesis.

The results obtained in this work show that glucose has a regulatory effect on the expression of several genes in *X. dendrorhous*, as has been shown in other yeasts. The mRNA levels of the *grg2 *gene decreased dramatically when glucose was added to the culture. Moreover, the *PDC *gene was induced by glucose, as it is in the majority of phylogenetically related organisms [22-25]. In addition, we found that adding glucose to the media caused a decrease in the mRNA levels of all of the carotenogenesis genes involved in the synthesis of astaxanthin from GGPP. In the majority of these experiments, the effect of glucose reached its maximum between 2 and 4 h after addition. By 24 h after glucose addition, mRNA levels returned to baseline. No data were collected between 6 and 24 h after the addition of the sugar, but in most cases the recovery was estimated to occur completely within the first 8 h after the addition of glucose. Furthermore, the remaining glucose determinations showed that the kinetics of sugar consumption was slower than the return to basal gene expression levels. This finding suggests some type of adaptation mechanism, which over time diminishes the transcriptional response to the presence of glucose.

The global effect of glucose on the carotenogenesis pathway may be related to the presence of binding sites for the MIG1 general catabolic repressor in the promoter regions of the *crtS *[7], *crtYB *and *crtI *genes [32]. Such sites are also present in the promoter region of the *grg2 *gene (unpublished data), suggesting that a homolog of the MIG1 regulator may mediate the glucose repression of these genes. However, further studies are needed to demonstrate the functionality and importance of these elements.

Interestingly, the repressive effect of glucose on *crtYB *and *crtI *is manifested in different ways on the alternative and mature transcripts of these genes. Considering that both transcripts of each gene come from a single transcriptional unit, their different expressions suggest the involvement of post-transcriptional regulatory mechanisms. Thus, the differential effect may be due to modification of the factors that control the alternative processing of these genes. This differential effect is in addition to previous observations that the amounts of the mature and alternative mRNAs for both genes vary during yeast growth, depending on the carbon source used, the age of the culture and the carotenoid content [10]. The functions of the *crtYB *and *crtI *alternative transcripts are unclear [10,15,32], although it has been established that they are generated from anomalous splicing of the respective non-processed messenger. The alternative mRNA of the *crtI *gene conserves 80 bp of the first intron, while the alternative mRNA of the *crtYB *gene conserves 55 bp of the first intron and lacks 111 bp of the second exon. In both cases, the alternate splice results in mRNAs with several premature stop codons in their sequences [10], suggesting that the alternative transcripts may not encode functional proteins. Studies performed in our laboratory indicate that mutant strains that only express the alternative mRNA of the *crtI *gene are unable to synthesize astaxanthin and they accumulate phytoene [33], indicating that this mRNA does not encode a functional phytoene desaturase protein. Considering these observations, the biological significance of the glucose-mediated repression of the alternative *crtYB *and *crtI *mRNAs is not clear.

An important observation is that the glucose-mediated repression of the *crtYB*, *crtI *and *crtS *genes was seriously compromised in mutant strains incapable of synthesizing astaxanthin. This observation is consistent with previous reports that showed that a decrease in astaxanthin content causes an increase in the total amount of carotenoids, suggesting that astaxanthin may have a negative feedback effect on pigment synthesis [27]. The results reported here indicate that an inability to synthesize astaxanthin would cause deregulation of a significant number of genes involved in the late stages of the pathway, thereby releasing it from repression by glucose and even increasing the availability of the messengers necessary for pigment synthesis.

By studying the effects of glucose on cell growth and early pigment production, we found that glucose promoted a high biomass production after 24 h, but completely inhibited carotenoid biosynthesis. Similar results were observed when other glucose-derived carbon sources were used, such as maltose and galactose (data not shown).

The early glucose-mediated inhibition of carotenoid synthesis can be explained, at least partially, by the decrease in the mRNA levels of the carotenogenesis genes. A previous study showed that overexpression of *crtYB *causes an increase in the amount of pigments produced and that overexpression of *crtYB *and *crtI *cause a change in the relative composition of the carotenoids synthesized [31]. These results indicate that changes in the mRNA levels of the carotenogenesis genes have a direct effect on pigment biosynthesis, supporting the importance of gene expression in the regulation of the pathway. However, it was not possible to establish *a priori *whether the inhibition of pigment synthesis caused by the addition of glucose was due only to a decrease in the expression of the carotenogenesis genes. Previous work has shown that high glucose concentrations (between 20 and 80 g/l) cause a reduction in the *X. dendrorhous *growth rate; the low carotenoid production may be associated with that inhibition [34]. However, our results indicate that, at least under the conditions tested, glucose can induce an important increase in biomass. Thus, the inhibition of carotenoid biosynthesis reported here cannot be explained by a reduction in growth rate. Another possibility is that the inhibition of pigment production is a consequence of the cell growth promoted by glucose, in contrast with the lack of growth observed in the control culture. However, the experiments were designed to evaluate the effect of glucose and ethanol over a short period of time after the addition of the carbon source (only during the first six hours). In most cases, the maximum effect on carotenogenic gene expression was observed between 2 and 4 h after the treatment; during this time, biomass variations were very low. In addition, *X. dendrorhous *exhibited very poor growth in other carbon sources, such as galactose, sorbose and succinate, registering a growth level equivalent to the control condition (data not shown), preventing these carbon sources from being used as a "growing" control.

It is well known that glucose has a global effect on cell metabolism, causing induction of genes related to glycolysis and fermentative metabolism and thereby repressing many of the genes involved in secondary metabolism and the use of alternative carbon sources [35]. The induction of the *PDC *gene and repression of the invertase-coding gene *INV *(data not shown) in response to glucose addition suggests that this phenomenon may also occur in *X. dendrorhous*. Therefore, the inhibition of pigment synthesis in response to glucose may also be a consequence of inhibition of the components of respiratory metabolism, which control the availability of substrates for the carotenogenesis pathway.

However, in contrast to glucose, non-fermentable carbon sources generally cause an increase in the synthesis of pigments in *X. dendrorhous *[12,13]. Accordingly, our results indicate that the addition of ethanol causes an increase in the total amount of carotenoids 24 h after treatment. Strikingly, even when there was an increase in the biomass, ethanol induced *de novo *synthesis of pigments, as evidenced by an increase in the relative amounts of intermediate carotenoids in the pathway. These results agree with a previous report by Gu and coworkers, in which the addition of ethanol at different stages of growth caused an increase in the total amount of carotenoids [14]. The authors proposed two mechanisms by which ethanol induced the biosynthesis of pigments. Initially, ethanol and its subsequent conversion into acetate by the aldehyde oxidase enzyme may have generated superoxide radicals, resulting in the induction of carotenoid synthesis. This outcome is consistent with previous work examining the induction effects of some reactive oxygen species on carotenogenesis [27,36,37]. Alternatively, acetate may have continued along its metabolic pathway towards the generation of acetyl coenzyme A, with the latter becoming the substrate for the synthesis of isoprenoids by the mevalonate pathway. This outcome is in agreement with previous reports demonstrating that the addition of mevalonate [38] and several other non-fermentable carbon sources [12,29] causes an increase in pigmentation production in *X. dendrorhous*, probably because of their direct conversion into isoprenoid precursors. Our results suggest that there is a possible third mechanism underlying increased pigmentation production, which is mediated by the increase in expression of the *crtYB *and *crtS *genes caused by the addition of alcohol. The increase in pigment synthesis mediated by ethanol is likely due to a combination of these proposed mechanisms as well as other factors not yet elucidated.

## Conclusion

The carbon source regulation of carotenoid biosynthesis in *X. dendrorhous *involves changes at the mRNA level of several genes. In the presence of glucose, the three genes involved in the synthesis of astaxanthin from GGPP were down-regulated, while *de novo *synthesis of pigments was inhibited. In contrast, ethanol caused early induction of carotenoid biosynthesis, which was correlated with induction of *crtYB *and *crtS *gene expression. Importantly, these results provide the first molecular explanation for the strong repression of carotenoid production observed when this yeast is grown in the presence of glucose.

## Methods

### Strains and culture conditions

The wild-type *X. dendrorhous *strain UCD67-385 was used for all experiments. Unless otherwise specified, the yeast cells were grown at 22°C with constant swirling (180 rpm/min) in YM liquid medium (0.3% yeast extract, 0.3% malt extract, 0.5% peptone) with or without glucose. The non-astaxanthin-producing strains used were the homozygous mutants T-YBHH2 (*crtYB^-^*), T-I21H1H (*crtI*^-^) and T-SHH2 (*crtS^-^*), described in a previous work [28]. These strains accumulate the carotenoid intermediates GGPP, phytoene and β-carotene, respectively. Yeast cell growth was assessed by measuring the optical density at 600 nm. For the determination of specific carotenoids, biomass was assessed by measuring the dry weight from 10-ml culture samples on an analytical balance (Shimadzu).

### RNA extraction and single strand DNA synthesis

To measure relative gene expression at different times and under different conditions, 40-ml culture aliquots were collected and centrifuged at 4000 × g, and the supernatants were discarded. The cell pellets were frozen in liquid nitrogen and stored at -80°C until use. Total RNA was extracted from the pellets by mechanical rupture of the cells, followed by addition of Tri-Reagent (Ambion) and shaking in a vortex apparatus with 0.5 mm glass beads (BioSpec) for 10 min. The lysates were then incubated for 10 min at room temperature, after which 150 μl of chloroform was added per ml of Tri-Reagent used. The mixtures were then centrifuged for 5 min at 4000 × g. For each sample, the aqueous phase was recovered and transferred to a clean RNase-free test tube. After two consecutive extraction cycles with acidic phenol:chloroform (1:1) and centrifugation at 4°C for 5 min, the RNA was precipitated by adding two volumes of isopropanol and incubating at room temperature for 10 min. Once precipitated, the RNA was washed with 75% ethanol, suspended in RNase-free H_2_O and quantified by determination of the absorbance at 260 nm in a double beam Shimadzu UV-150-20 spectrophotometer.

The synthesis of cDNA was performed using 5 μg of total RNA, 1.25 μM oligo-dT^18 ^primer, 0.5 μM dNTPs and 200 units of M-MLV reverse transcriptase (Invitrogen) in a final volume of 20 μl, according to the enzyme manufacturer's recommended protocol.

### Quantitative RT-PCR

Relative mRNA expression levels were determined in a Mx3000P quantitative PCR system (Stratagene) using 1 μl of the reverse transcription reaction, 0.25 μM of each primer and 10 μl of the SensiMix SYBR Green I (Quantace) kit in a final volume of 20 μl. The primers used to determine the relative levels of expression are detailed in Table 1. All of the primer pairs used to amplify each gene had efficiencies greater than 95%, as determined by standard curves, with correlation coefficients (R^2^) ≥ 0.996.

**Table 1:** Primers used in this work

Primer	Gene	Direction	Sequence (5' to 3')	Location
**mactF-RT**	*act*	F	CCGCCCTCGTGATTGATAAC	Spanning exons 2 & 3
**mactR-RT**	*act*	R	TCACCAACGTAGGAGTCCTT	Spanning exons 4 & 5
**mmcrtYBF2-RT**	*crtYB*(mm)	F	TCGCATATTACCAGATCCATCTGA	Spanning exons 1 & 2
**mmcrtYBR2-RT**	*crtYB*(mm)	R	GGATATGTCCATGCGCCATT	Exon 2
**amcrtYBF-RT**	*crtYB*(am)	F	GTGTGCATATGTGTTGCAACCA	Spanning exon 1 & intron 2
**amcrtYBR-RT**	*crtYB*(am)	R	AGAAGGTGCCTAGTTGCCAAGA	Exon 3
**mmcrtIF-RT**	*crtI*(mm)	F	CATCGTGGGATGTGGTATCG	Spanning exons 1 & 2
**mmcrtIR-RT**	*crtI*(mm)	R	GGCCCCTGATCGAATCGATAA	Spanning exons 3, 4, 5
**amcrtIF-RT**	*crtI*(am)	F	CGTGGTTTAATCCGTATCAGC	Spanning exon 1 & intron 1
**amcrtIR2-RT**	*crtI*(am)	R	TCTCGAACACCGTGACCT	Exon 2
**mcrtSF-RT**	*crtS*	F	ATGGCTCTTGCAGGGTTTGA	Spanning exons 6 & 7
**mcrtSR-RT**	*crtS*	R	TGCTCCATAAGCTCGATCCCAA	Spanning exons 8 & 9
**grg2real FW1**	*grg2*	F	CATCAAGACCTCTGTCACCAAC	Spanning exons 1 & 2
**grg2real RV1**	*grg2*	R	TTGGCGTCAGACGAGGACT	Exon 3
**pdcreal FW1**	*PDC*	F	TCAACACTGAGCTGCCCACT	Spanning exons 5 & 6
**pdcreal RV1**	*PDC*	R	ATTCCGAATCGGGAAGCACA	Exon 6

F: Forward, R: Reverse; (mm): mature transcript, (am): alternatively spliced transcript.

The Ct values obtained for each reaction were normalized to the respective value for the β-actin gene and were later expressed as functions of the control conditions using the ΔΔCt algorithm [39].

### Extraction and analysis of pigments

For the extraction of pigments, aliquots of cell cultures were collected under the different conditions tested. The aliquots were centrifuged at 4000 × g, and the supernatants were subsequently discarded. Each cell pellet was suspended in 2 ml of an acetone:water mixture (1:1), and 500 μl of 0.5-mm glass beads were then added. After 10 min of vortex shaking, the mixture was centrifuged at 4000 × g for 3 min. Next, the supernatant was transferred to a clean test tube, and 2 ml of acetone was added to the pellet. The tube containing the pellet was then vortex stirred for 3 min and centrifuged at 4000 × g for 3 min, after which the supernatant was collected and mixed with the supernatant that had been previously set aside. These steps were repeated until the recovered supernatant was completely colorless. The collected supernatants were then treated with 0.25 volumes of water and 0.25 volumes of petroleum ether; this mixture was mixed and centrifuged for 3 min at 4000 × g. Subsequently, the petroleum ether (top) phase was recovered, and its absorbance at 465 nm was determined. The pigment concentrations were quantified using the average of the molar extinction coefficients of astaxanthin and β-carotene (2346 cm^-1^/M).

The pigment composition was determined by RP-HPLC using a LiChrospher RP18 125-4 (Merck) column and an acetonitrile:methanol:isopropanol (85:10:5) mobile phase with a 1 ml/min flow rate under isocratic conditions. Each pigment was identified by comparison with specific standards (Sigma) based on their retention time and absorption spectra [40] using a Shimadzu SPD-M10A diode array detector.

### Quantification of glucose in the extracellular medium

The glucose present in the extracellular medium was quantified by determining the increase in absorbance at 340 nm due to the production of NADPH as a product of the oxidation of the glucose present, using the D-Glucose/D-fructose kit (Megazyme).

## Authors' contributions

AM and MN participated in the design of the study, conducted the transcriptional repression analysis of the genes involved in the synthesis of astaxanthin and cloned the *grg*2 and *PDC *genes. AW and CL conducted the pigment analysis. JA participated in the construction of mutant strains. MB participated in the study design. VC conceived this work and participated in its design and coordination. All authors read and approved the final manuscript.
